# Quantitative Analyses of the Yeast Oxidative Protein Folding Pathway *In Vitro* and *In Vivo*

**DOI:** 10.1089/ars.2018.7615

**Published:** 2019-06-24

**Authors:** Dave M. Beal, Emma L. Bastow, Gemma L. Staniforth, Tobias von der Haar, Robert B. Freedman, Mick F. Tuite

**Affiliations:** ^1^ Kent Fungal Group, School of Biosciences, University of Kent, Canterbury, United Kingdom.; ^2^ School of Life Sciences, Gibbet Hill Campus, University of Warwick, Coventry, United Kingdom.

**Keywords:** disulfide bond, endoplasmic reticulum, protein disulfide isomerase, ERO1

## Abstract

***Aims:*** Efficient oxidative protein folding (OPF) in the endoplasmic reticulum (ER) is a key requirement of the eukaryotic secretory pathway. In particular, protein folding linked to the formation of disulfide bonds, an activity dependent on the enzyme protein disulfide isomerase (PDI), is crucial. For the *de novo* formation of disulfide bonds, reduced PDI must be reoxidized by an ER-located oxidase (ERO1). Despite some knowledge of this pathway, the kinetic parameters with which these components act and the importance of specific parameters, such as PDI reoxidation by Ero1, for the overall performance of OPF *in vivo* remain poorly understood.

***Results:*** We established an *in vitro* system using purified yeast (*Saccharomyces cerevisiae*) PDI (Pdi1p) and ERO1 (Ero1p) to investigate OPF. This necessitated the development of a novel reduction/oxidation processing strategy to generate homogenously oxidized recombinant yeast Ero1p. This new methodology enabled the quantitative assessment of the interaction of Pdi1p and Ero1p *in vitro* by measuring oxygen consumption and reoxidation of reduced RNase A. The resulting quantitative data were then used to generate a simple model that can describe the oxidizing capacity of Pdi1p and Ero1p *in vitro* and predict the *in vivo* effect of modulation of the levels of these proteins.

***Innovation:*** We describe a model that can be used to explore the OPF pathway and its control in a quantitative way.

***Conclusion:*** Our study informs and provides new insights into how OPF works at a molecular level and provides a platform for the design of more efficient heterologous protein expression systems in yeast.

## Introduction

The oxidative protein folding (OPF) pathway of the endoplasmic reticulum (ER) has been well characterized in the yeast *Saccharomyces cerevisiae*, both in terms of identification of components and defining how they interact. The key components of the pathway are protein disulfide isomerase in yeast (Pdi1p) (13) and ER oxidase in yeast (Ero1p) (16, 41), encoded by the essential *PDI1* and *ERO1* genes, respectively.

Biochemical, structural, and genetic characterization of the pathway have demonstrated that it comprises a linear electron transfer pathway. Electrons from the thiol (SH) groups of reduced proteins flow *via* Pdi1p to Ero1p through a series of thiol:disulfide oxidoreductions, to a bound flavin adenine dinucleotide (FAD) group within Ero1p, and thence to molecular oxygen (O_2_). The net result of this process is the formation of disulfide (S-S) bonded proteins and hydrogen peroxide (18, 19, 55, 56).

Given the similarities in the core components of the yeast and human OPF systems, it is not surprising that some functional complementation of components between the species has been observed both *in vivo* (38) and *in vitro* (4). Despite this, there are marked differences between species in the number of members of the protein disulfide isomerase (PDI) and endoplasmic reticulum oxidase (ERO) families, in their regulation and in the associated components of the pathway.

Innovation Oxidative protein folding (OPF) in eukaryotes utilizes two proteins, protein disulfide isomerase (PDI) and endoplasmic reticulum oxidase (ERO), to generate correctly positioned disulfide bonds. Using an advanced *in vitro* refolding process to isolate functional recombinant yeast ERO (Ero1p), we carried out the first quantitative assessment of the rates of PDI/ERO oxidation by oxygen. Based on these quantitative data, we developed an ordinary differential equations-based model of the oxidizing capabilities of the yeast endoplasmic reticulum *in vivo*. This model gives new insights into native yeast OPF and suggests that engineering strategies to enhance the secretion of authentic high-value recombinant proteins from yeast need to consider an expanded parameter space comprising both Pdi1p and Ero1p, depending on the efficiency with which Pdi1p and the recombinant substrate interact.

Yeast and human ERO proteins show functional identity but also significant structural differences, especially in the number and arrangement of nonactive-site disulfide bonds (18, 25). These disulfides play regulatory roles that differ considerably between the species, although both in yeast and in human systems, the formation and breakage of regulatory disulfides in ERO are critically catalyzed by PDI (1–3, 5, 7, 8, 28, 43). Recently it has been proposed that the mode of regulation of OPF differs between humans and yeast in that the human pathway provides for clear on/off switching, whereas regulation in the yeast system is more graduated (37, 66).

In *S. cerevisiae*, a simple linear pathway operates and both *ERO1* and *PDI1* are essential genes. However, viability of the *pdi1* deletion strain can be restored by overexpression of a number of alternative PDI-like genes such as *EUG1* (52). In contrast, human cells lacking both ERO genes (α and β) are viable, in part, because other proteins are available in the ER that can function as electron acceptors from PDI. These include the peroxidases GPX7 and GPX8 and possibly the peroxiredoxin PrxIV, which can use hydrogen peroxide (H_2_O_2_) generated by ERO or other systems, as terminal electron acceptor (26, 36, 42, 53, 59, 67). Such activities are lacking in the yeast ER. Furthermore, the human pathway is also more complex in that a large number of PDI family members can interact with ERO in either a regulatory or catalytic electron transfer role (34, 50).

**FIG. 1.**
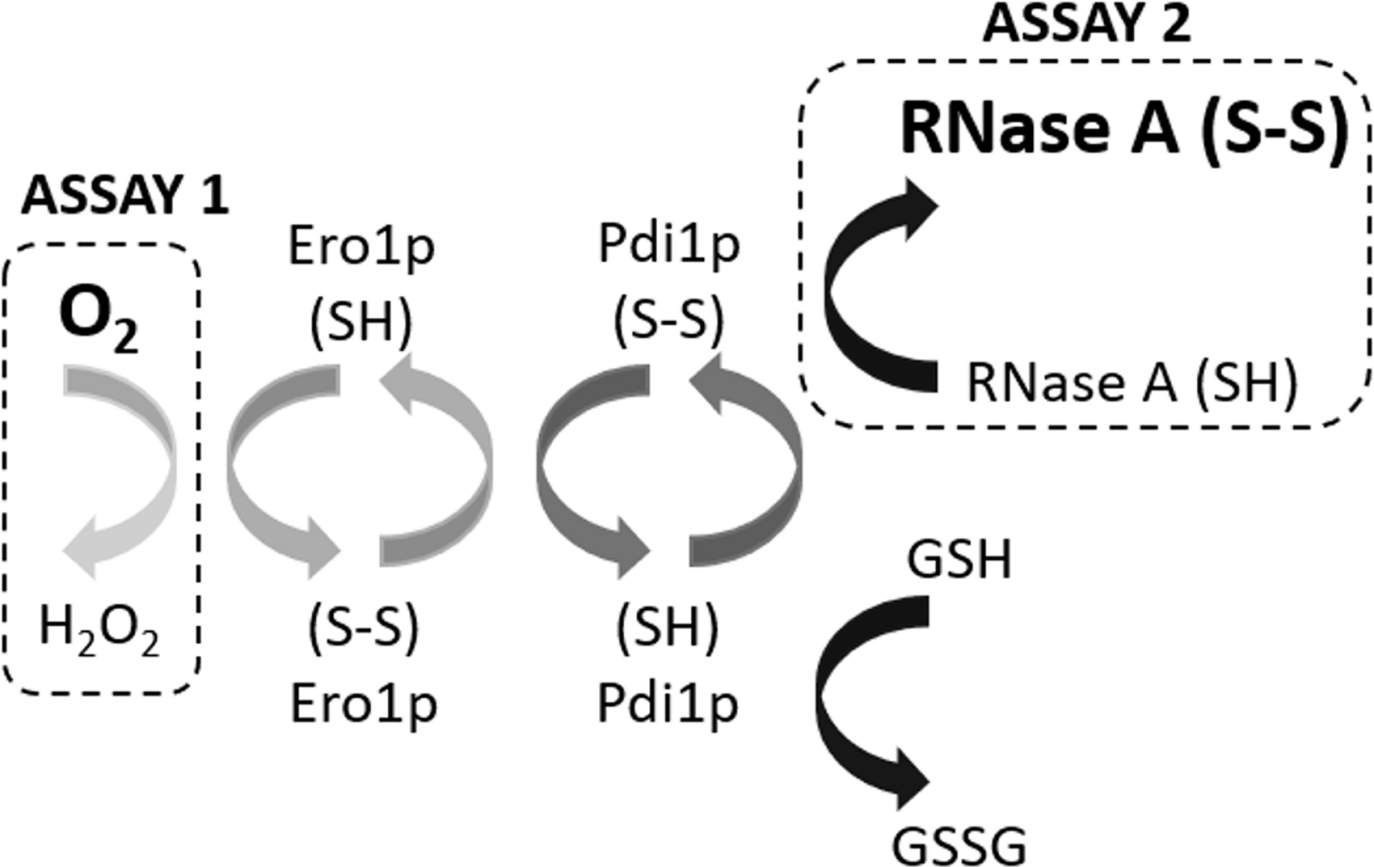
**Methods for *in vitro* analysis of electron flow in the yeast OPF pathway.** The ER oxidative folding pathway can be reconstituted with Ero1p and Pdi1p in the presence of dissolved O_2_ as the terminal electron acceptor and either GSH (Assay 1) or reduced protein substrate (Assay 2) as the electron source. The operation of the pathway can be monitored either by the disappearance of O_2_ (Assay 1), by the change in redox state of the substrate protein (Assay 2). ER, endoplasmic reticulum; Ero1p, purified yeast endoplasmic reticulum oxidase 1; GSH, glutathione; GSSG, glutathione disulfide; H_2_O_2_, hydrogen peroxide; O_2_, molecular oxygen; OPF, oxidative protein folding; Pdi1p, purified yeast protein disulfide isomerase.

To better understand the OPF pathway and its regulation, PDI-dependent electron transfer has been effectively reconstituted *in vitro* using purified proteins where pathway activity can be assayed by monitoring formation of disulfide-bonded proteins (30, 54) or consumption of O_2_ (27, 57, 58) (Fig. 1).

Disulfide-bonded proteins including antibodies, cytokines, and serum proteins are valuable biopharmaceuticals, and *S. cerevisiae* remains an attractive, safe, and low-cost host cell in which to generate them (47, 65).

Many of these high-value biopharmaceuticals contain inter- and/or intramolecular disulfide bonds and thus need to transit through the ER to be correctly folded. However, the secretory capacity of *S. cerevisiae* is low and has to deal with many fewer secreted proteins than a mammalian cell, for example. As a result, there have been attempts to engineer OPF to increase the production of such high-value disulfide-bonded targets. Indeed, overexpression of yeast and human PDI in yeast was shown at an early stage to improve the production of a range of recombinant disulfide-bonded proteins (44, 47), and this strategy is exploited industrially in the production of human serum albumin and transferrin, for example (14, 40).

While showing a degree of success in terms of improving product yield and/or authenticity, interventions intended to increase the yield of the pathway in production and secretion of recombinant proteins can have unexpected outcomes [see *e.g.*, de Ruijter *et al.* (45)]. A key issue is that knowledge of kinetic properties of the components and quantitative analyses of the pathway are lacking and this deficit has discouraged systems biology approaches to rational engineering of the OPF pathway (57). In this study, we have reconstituted the OPF of *S. cerevisiae in vitro* and obtained the first quantitative data on the kinetics of the overall system and developed a model capable of predicting how the system can be modified to enhance OPF. Such information will underpin future design strategies for enhanced secretion of high-value complex biopharmaceuticals from this simple eukaryote.

## Results

### Production of active recombinant Pdi1p and Ero1p

**FIG. 2.**
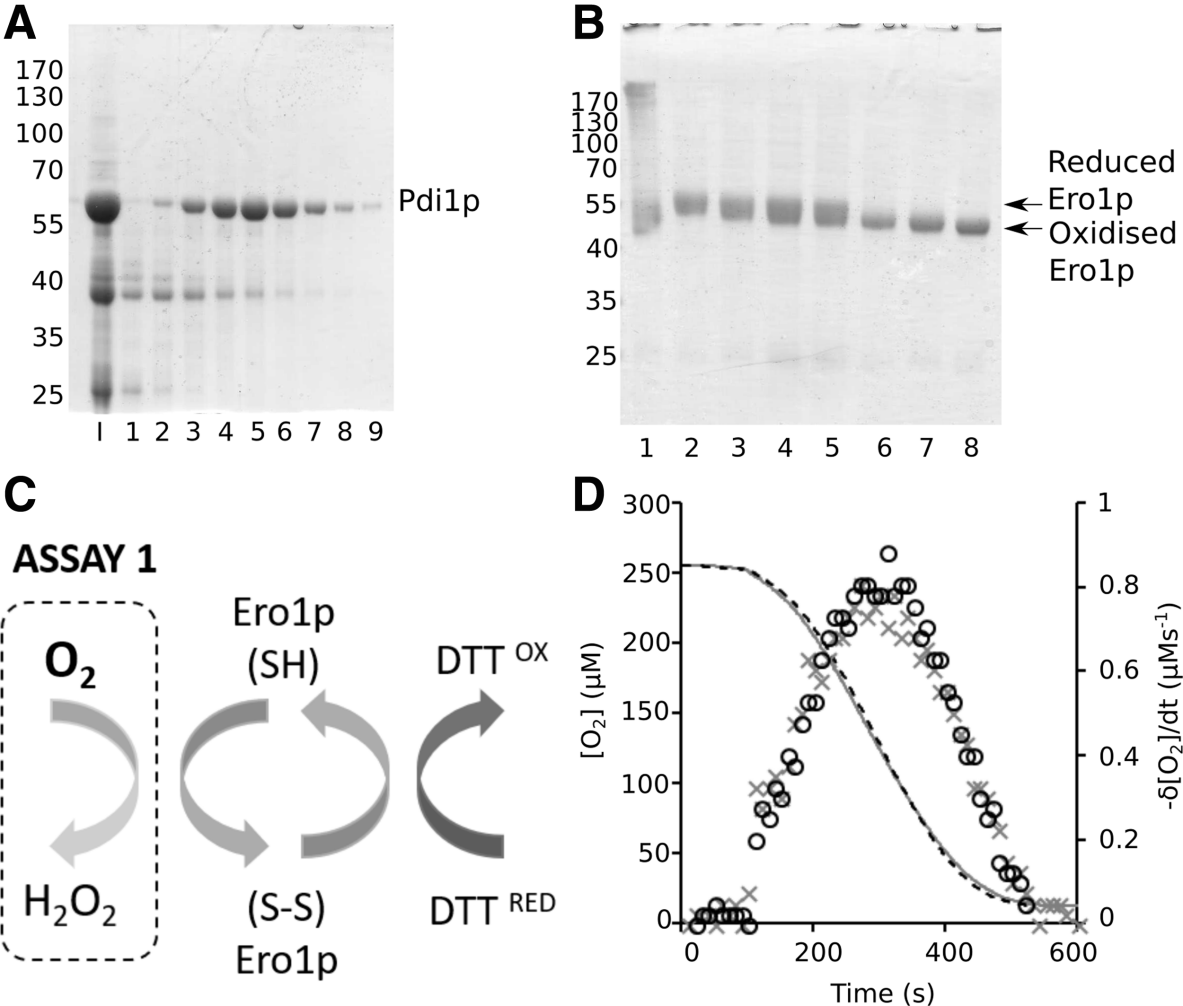
**Purification of proteins for analysis of the yeast OPF pathway. (A)** Reducing SDS-PAGE of Pdi1p purification (I) input, lanes 1–9; fractions from HiTrap Q FF purification. **(B)** Reducing SDS-PAGE of Ero1p purification after AMS trapping: Lane 1, post-Ni-NTA purification; lane 2, reduction of the Ni-NTA eluent by DTT (30 m*M*); lanes 3–6, reoxidation in the presence of FAD at 10 min, 30 min, 60 min, and 18 h; lane 7, purified Ero1p trapped with AMS; lane 8, purified Ero1p trapped with NEM. **(C)** Schematic showing the Ero1p activity assay where Ero1p reduced by DTT is reoxidized by O_2_, which is measurable using a Clarke electrode. **(D)** O_2_ consumption assay using a Clarke electrode. One micromolar Ero1p equilibrated for 100 s before addition of DTT (10 m*M* final concentration) to initiate Ero1p-mediated O_2_ consumption (primary axis). The graph displays two replicates of the experiment (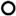 and 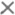), respectively. The nonlinearity of O_2_ consumption is shown by the increase in rate of reaction overtime to a maximum before decreasing again to 0 (secondary axis). AMS, 4-acetamido-4′-maleimidylstilbene-2,2′-disulfonic acid; DTT, 1,4-dithiothreitol; FAD, flavin adenine dinucleotide; Ni-NTA, Ni^2+^ loaded nitrilotriacetic acid modified sepharose; SDS-PAGE, sodium dodecyl sulfate-polyacrylamide gel electrophoresis.

To date, biochemical studies using Pdi1p have relied on bacterially produced protein, which is biochemically active (29, 30, 37, 57). In native environments, a 21-residue N-terminal signal sequence directs Pdi1p to the ER where it is then cleaved to give the mature and functional protein. To mimic functional Pdi1p and increase protein yield in bacterial expression systems, it is common to remove the signal sequence from the construct and replace it with a polyhistidine tag (30, 54, 57). We used this strategy to obtain highly pure Pdi1p_22-530_, lacking the signal sequence but containing an N-terminal six-histidine tag (Fig. 2A).

In contrast to Pdi1p, Ero1p does not express well as a full-length construct and consequently, previous studies have relied on truncated Ero1p constructs (10, 18). We chose to use the longest Ero1p construct we could express in reasonable yield in *Escherichia coli* without mutation of cysteine residues (Ero1p_19-424_), again with an N-terminal six-histidine tag to facilitate purification.

The resulting purified Ero1p_19-424_ existed as a mixture of oxidation products postpurification. To yield homogeneously oxidized Ero1p_19-424_ containing stoichiometric amounts of FAD, we exploited a controlled sequence of reduction and oxidation in the presence of FAD (Fig. 2B). Using an assay for O_2_ consumption in a 1,4-dithiothreitol (DTT)-dependent manner (Fig. 2C), we were able to demonstrate that the Ero1p_19-424_ reproducibly consumed O_2_ with general kinetic characteristics as reported by others (37, 57) (Fig. 2D). Our homogeneously oxidized Ero1p_19-424_ had a maximum rate of 0.8 μ*M* O_2_ per μ*M* Ero1p per second when treated with 10 m*M* DTT.

### Rate of thiol oxidation is linearly dependent on Pdi1p and Ero1p concentrations

**FIG. 3.**
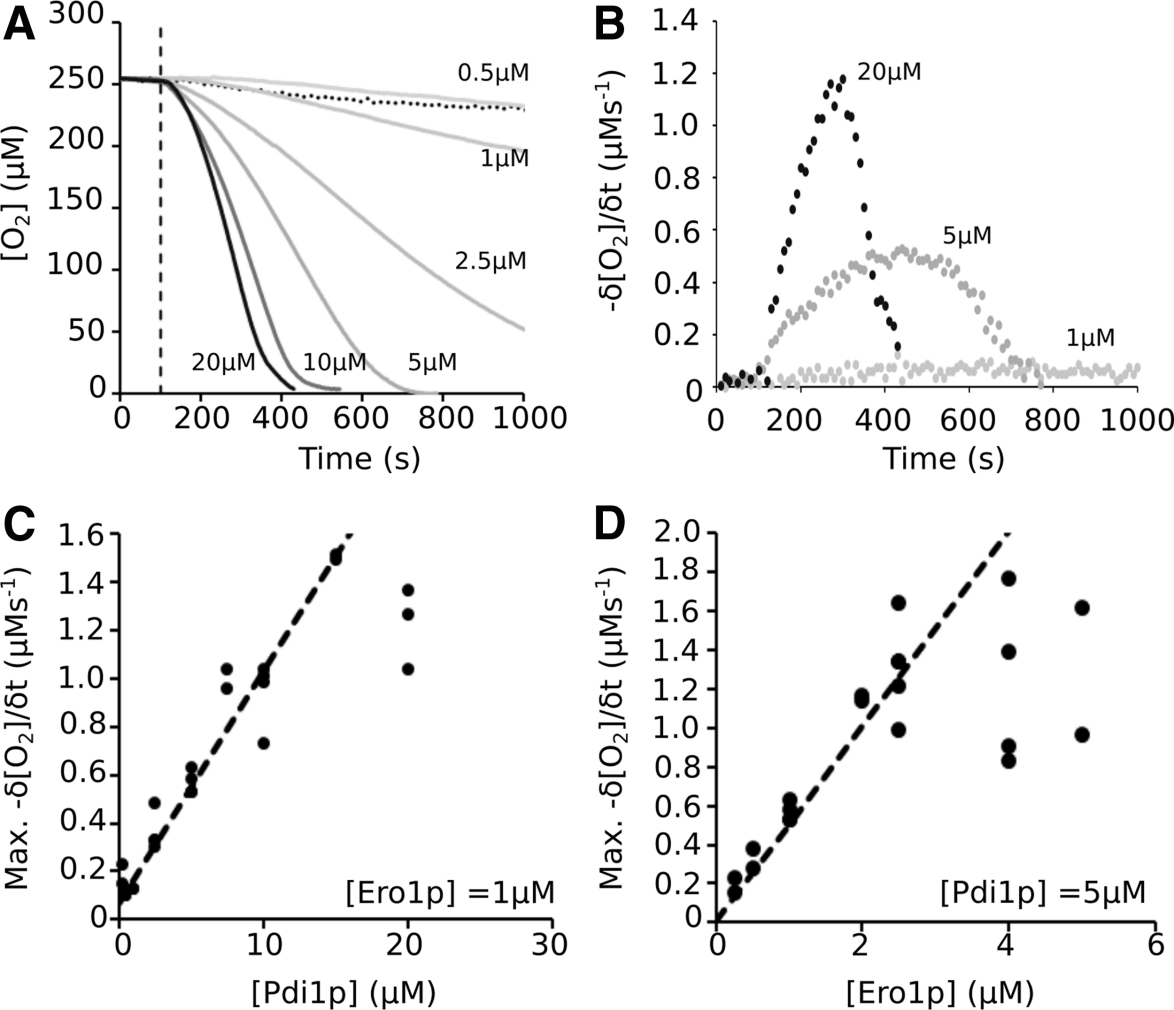
**The oxidizing activity of the yeast OPF pathway is dependent on the concentration of Pdi1p and Ero1p. (A)** The O_2_ consumed when variable concentrations of Pdi1p are reoxidized by Ero1p (1 μ*M*) in the presence of 40 m*M* GSH. Buffer composition: 100 m*M* Tris-AcOH, 50 m*M* NaCl, pH 8, 25°C. All reagents except the Ero1p were combined and equilibrated, with constant stirring, to give a static baseline before Ero1p addition 100 s after the initiation of the experiment. An average of at least two replicates is plotted. **(B)** The nonlinearity of the observed O_2_ consumption was assessed by determining the change in rate of O_2_ consumption over the course of the experiment, using 10 s intervals. Three representative curves are shown (20, 5, and 1 μ*M*), all exhibiting a slow increase in rate toward a maximum value before decreasing. (**C, D)** The extracted maximum rates of O_2_ consumption for all repeats, as determined from the data in **(B)**, and plotted against enzyme concentration for Pdi1p **(C)** and Ero1p **(D)**. AcOH, acetic acid; NaCl, sodium chloride.

Glutathione (GSH)-mediated O_2_ consumption (Fig. 1, Assay 1) was used to study the limits of the coupled system using variable Ero1p and Pdi1p concentrations. At a fixed 1 μ*M* concentration of Ero1p, the rate of O_2_ consumption was dependent on the concentration of Pdi1p (Fig. 3A, B). This dependence was linear at concentrations <15 μ*M* before the system saturated (Fig. 3C). Conversely, at a fixed 5 μ*M* concentration of Pdi1p, the maximum rate was linearly dependent on Ero1p at concentrations up to 2 μ*M* (Fig. 3D). In both cases, the maximum rate of O_2_ consumption observed in saturating conditions was ∼1.5 μ*M*/s.

Although GSH is present within the ER, the physiological substrates of Pdi1p are proteins. A frequently used model substrate *in vitro* is reduced and denatured ribonuclease A (rdRNase A) (30, 54), a single chain protein containing four intrachain disulfide bonds. In the GSH-dependent assay of the Pdi1p/Ero1p interaction, the electron donor, GSH, is present in vast excess. When GSH is replaced as an electron source by rdRNase A, the number of electrons available to the system is limited by the solubility of rdRNase A.

**FIG. 4.**
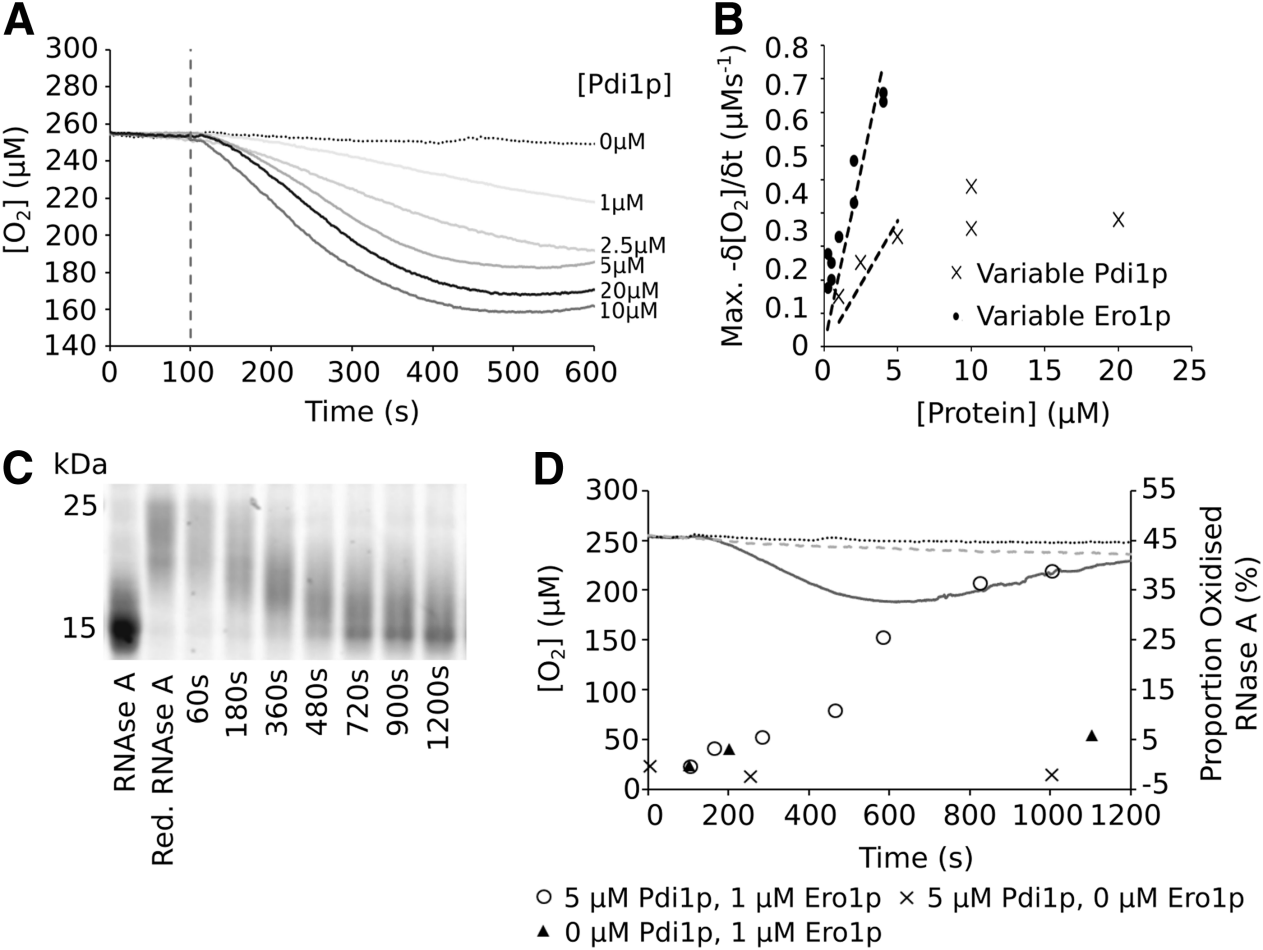
**The interaction of Pdi1p and Ero1p mediates reoxidation of rdRNase A. (A)** O_2_ consumption observed when 60 μ*M* rdRNase A was added to Pdi1p and Ero1p. The concentration of Pdi1p was varied between 1 and 20 μ*M* with a fixed Ero1p concentration of 1 μ*M*. All reagents except Ero1p were combined and stirred at a constant speed for 100 s before addition of Ero1p. **(B)** The maximum rate of O_2_ consumption at different enzyme concentrations: variable Pdi1p with fixed [Ero1p] (1 μ*M*) (*dark gray ×* ) and variable Ero1p with fixed [Pdi1p] (5 μ*M*) (*black filled circles*). Data are shown for all replicates. **(C)** 15% Tris-Gly SDS-PAGE analysis of the Pdi1p (5 μ*M*)/Ero1p (1 μ*M*) mediated reoxidation of RNase A overtime using AMS trapping. **(D)** O_2_ consumption for RNase A reoxidation. The primary axis, for 5 μ*M* Pdi1p and 1 μ*M* Ero1p (*dark gray*), no Pdi1p (*dotted line*), and no Ero1p (*dashed line*). The secondary axis shows the quantitation, by Image J, of the oxidized fraction from **(B)** over the course of the experiment. ▴, no Pdi1p; × , no Ero1p; ○, 5 μ*M* Pdi1p and 1 μ*M* Ero1p. rdRNase A, reduced and denatured ribonuclease A; Tris-Gly, tris-glycine buffer.

Using the rdRNase A assay (Fig. 1, Assay 2), we found that at 60 μ*M* rdRNase A (*i.e.*, 240 μ*M* disulfides) and 1 μ*M* Ero1p, the maximum rate of O_2_ consumption was again linearly dependent on the concentration of Pdi1p, reaching saturation >10 μ*M* (Fig. 4A, B). Similarly, at 5 μ*M* Pdi1p, the rate was dependent on Ero1p concentrations (Fig. 4B). O_2_ consumption correlated with a change in redox state of the ribonuclease A (RNase A) (Fig. 4C and Supplementary Fig. S1), and the observed formation of disulfide bonds in the protein was clearly dependent on the presence of both Pdi1p and Ero1p (Fig. 4D). It is noteworthy that at a substrate:Pdi1p:Ero1p ratio of 60:5:1, the substrate can be fully reoxidized within 20 min in this assay.

### The pH dependence of the Pdi1p/Ero1p catalytic cycle

**FIG. 5.**
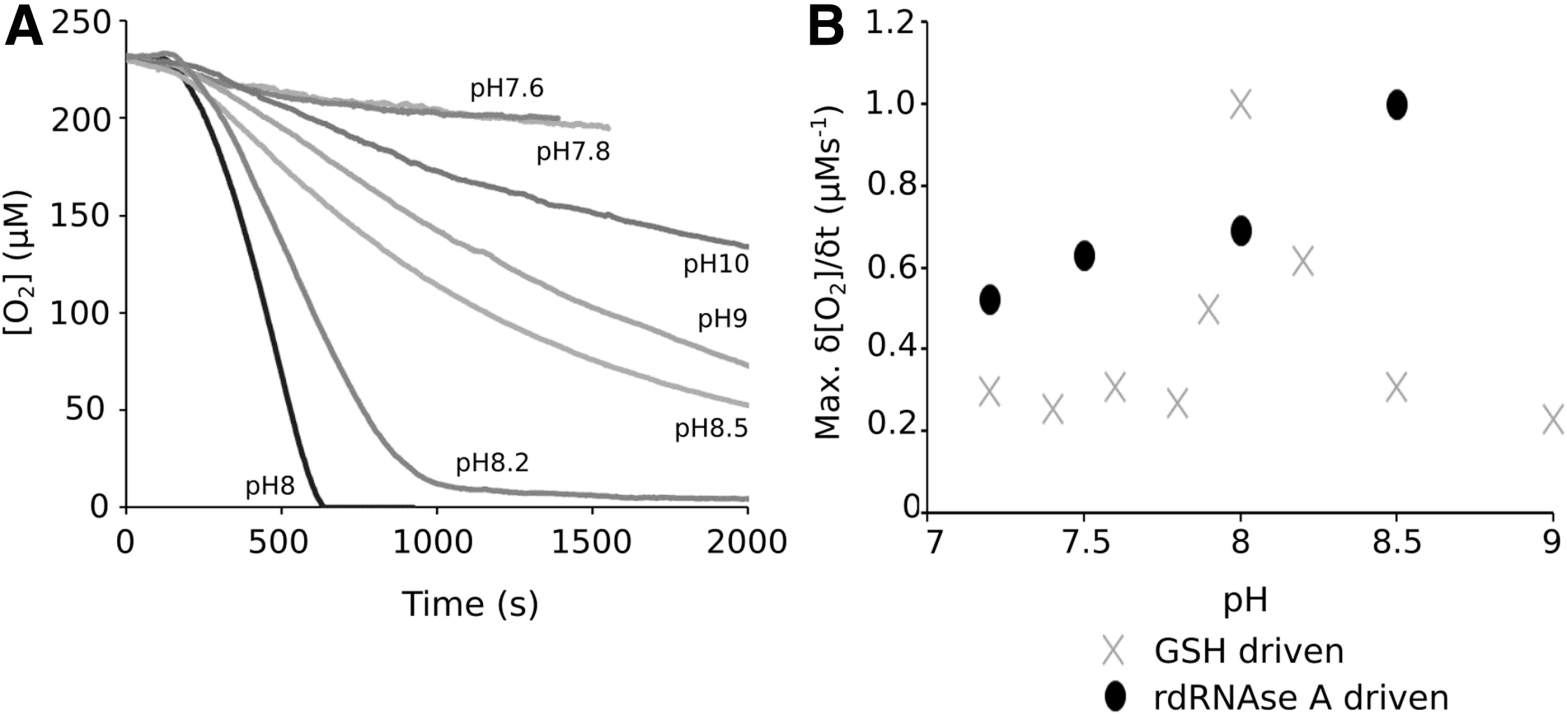
**GSH-mediated Pdi1p/Ero1p interaction is pH regulated. (A)** O_2_ consumption traces for 5 μ*M* Pdi1p and 1 μ*M* Ero1p when treated with 40 m*M* GSH under identical conditions except for variation of the reaction pH. **(B)** Comparison of the maximum rate of O_2_ consumption at different pH values with either GSH ( × ) or rdRNase A (●) as electron source.

To determine the optimum pH of the GSH-promoted Pdi1p and Ero1p catalytic cycle, the interaction of 5 μ*M* Pdi1p and 1 μ*M* Ero1p was assessed at different pH values (Fig. 5A, B). This analysis showed an extreme dependence on the pH of the reaction mixture for the GSH-driven system with a narrow window of optimum activity focused around pH 8. In contrast to this narrow activity range observed with GSH as the electron source, the system driven by 60 μ*M* RNase A showed broader tolerance to fluctuations in pH (Fig. 5B). We were unable to find reported values for the pH of the yeast ER, although for mammalian cells this has been reported as 7.5 (33, 39). Thus, under these pH conditions, proteins would be the preferred substrate for Pdi1p despite the presence of GSH.

### The relationship between Pdi1p oxidation state and refolding activity

**FIG. 6.**
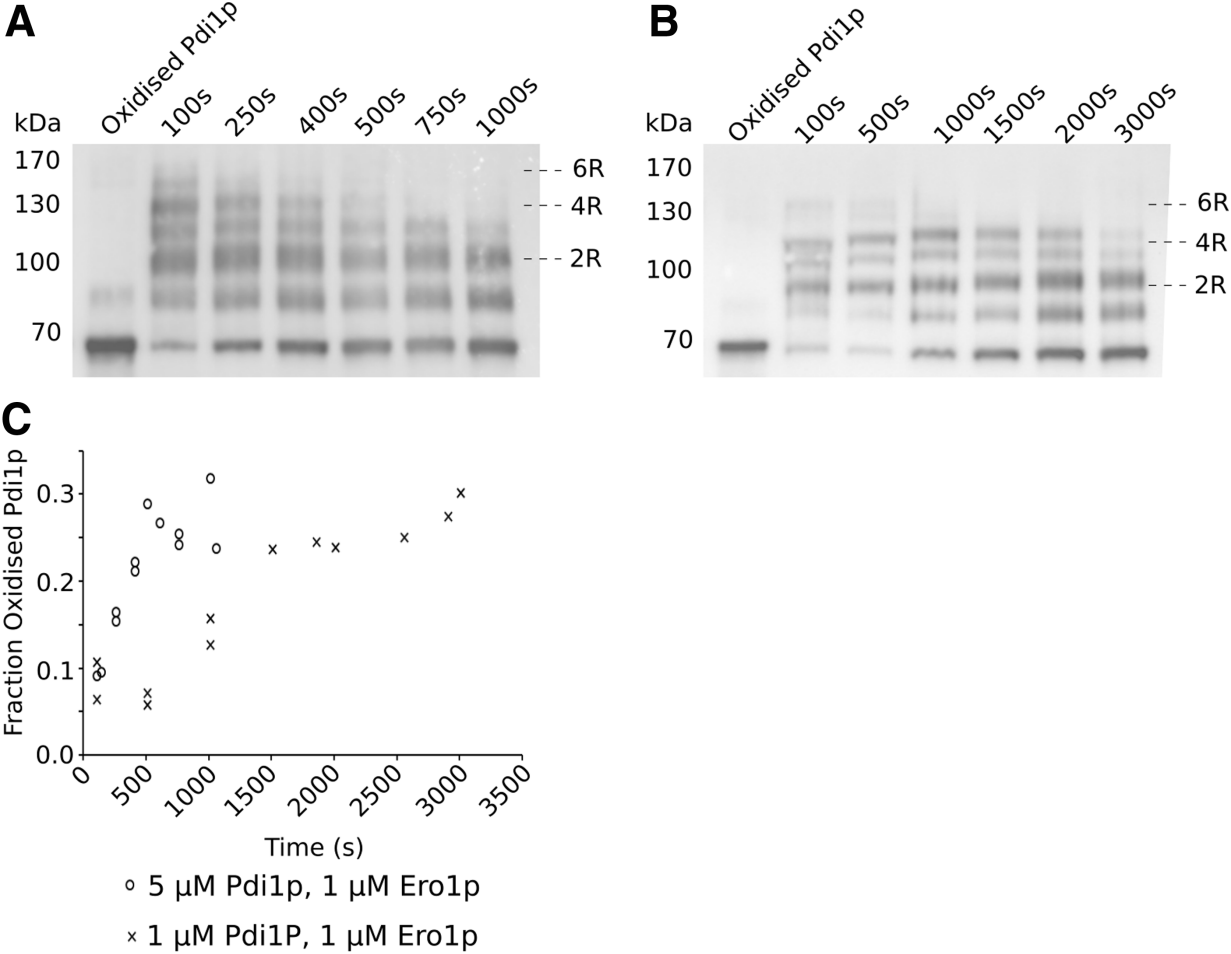
**The bulk Pdi1p redox state remains reduced when it becomes the limiting component. (A)** Anti-Pdi1p Western blot showing the redox state of Pdi1p over the course of Ero1p-mediated RNase A (60 μ*M*) reoxidation catalyzed by 5 μ*M* Pdi1p, 1 μ*M* Ero1p. **(B)** Anti-Pdi1p Western blot showing the redox state of Pdi1p over the course of Ero1p-mediated RNase A (60 μ*M*) reoxidation catalyzed by 1 μ*M* Pdi1p, 1 μ*M* Ero1p. **(C)** Densitometry analysis of the oxidized band over the course of the experiment for 5 μ*M* Pdi1p, 1 μ*M* Ero1p (○) and 1 μ*M* Pdi1p, 1 μ*M* Ero1p ( × ).

To examine in more detail how the internal oxidation state of Pdi1p relates to oxidative refolding activity in our *in vitro* system, a polyethylene glycol (PEG)-trapping assay was exploited that, through alkylation of reduced cysteines, leads to defined shifts in apparent molecular weight (48). Analysis of PEG-treated Pdi1p over the course of the oxidative refolding reaction by Western blotting showed that within 100 s of RNase A refolding, the initially fully oxidized Pdi1p becomes heavily reduced with a prominent species appearing at a size expected for four reduced cysteines, as well as a less abundant species corresponding to six reduced cysteines, that is, fully reduced Pdi1p (Fig. 6A, B; Supplementary Figs. S2 and S3).

Niu *et al.* (37) predicted that a fully reduced species of Pdi1p should form under strongly reducing conditions, which is the case for our *in vitro* system (Fig. 6A, B; Supplementary Figs. S2 and S3). This suggests that our assays operate in the high Ero1p activity regime proposed by Niu *et al.* (37). However, contrary to these data, we observed that the maximal activity of 5 μ*M* Pdi1p and 1 μ*M* Ero1p occurred between 400 and 600 s, when these more reduced components would have been depleted from the mixture (Fig. 6A and Supplementary Fig. S2). The presence of these more reduced forms of Pdi1p at the very beginning of the reaction rather than during the more active period of activity implies that reduction is a side effect of Ero1p activation rather than controlling the rate of Pdi1p activity.

Our data also showed that different ratios of Pdi1p to Ero1p consume O_2_ and, therefore, reoxidize RNase A at different rates (Fig. 4A, B). Comparison of the redox poise of the Pdi1p, the distribution of oxidation states, over the course of these reactions (Fig. 6A, B; Supplementary Figs. S2 and S3), showed that at 5:1 Pdi1p:Ero1p ratio, Pdi1p returns to a basal state of oxidation, consisting of fully oxidized Pdi1p but with a significant proportion with 1 reduced disulfide, much faster than an equimolar (1:1) ratio (Fig. 6C).

### Modeling oxidative folding

Our biochemical assays interrogate OPF from several different angles. We sought to unify the quantitative information from these assays by using an ordinary differential equations-based mathematical model of the biochemical reactions.

In establishing the model, we made several design decisions. For example, previous studies have shown that Pdi1p has asymmetric active sites with only one of the CGHC motifs responsible for the oxidase activity and the other being more important for isomerase activity (30). Consequently, to simplify modeling, we only utilized one Pdi1p active site in our model. Several reactions, such as the oxidation of Ero1p by molecular O_2_, are in reality multistep reactions. However, these are likely fast reactions, and as a consequence we modeled these as a single-step reaction. The reduction of oxidized Pdi1p by GSH likely occurs by sequential reaction with two consecutive GSH moieties, which are biochemically very similar. Because of their similarity we assumed that these reactions occur with identical rates. However, as we show hereunder, the rate constants for these reactions are not limiting for the time evolution of the model, so this has no consequences for deriving the remaining limiting rate constants.

In initial model runs, we also experimented with the inclusion of an inhibiting reaction between peroxide and Pdi1p, but since this did not alter the behavior of the model, we did not include this reaction in our final runs. We did, however, include an initial activation step involving the sequential reduction of two regulatory disulfides that yields active Ero1p. This step was introduced to account for the clear presence of a lag phase in our biochemical assays (Fig. 3A), but also to reflect the published biochemical evidence for such a step (37, 49).

**FIG. 7.**
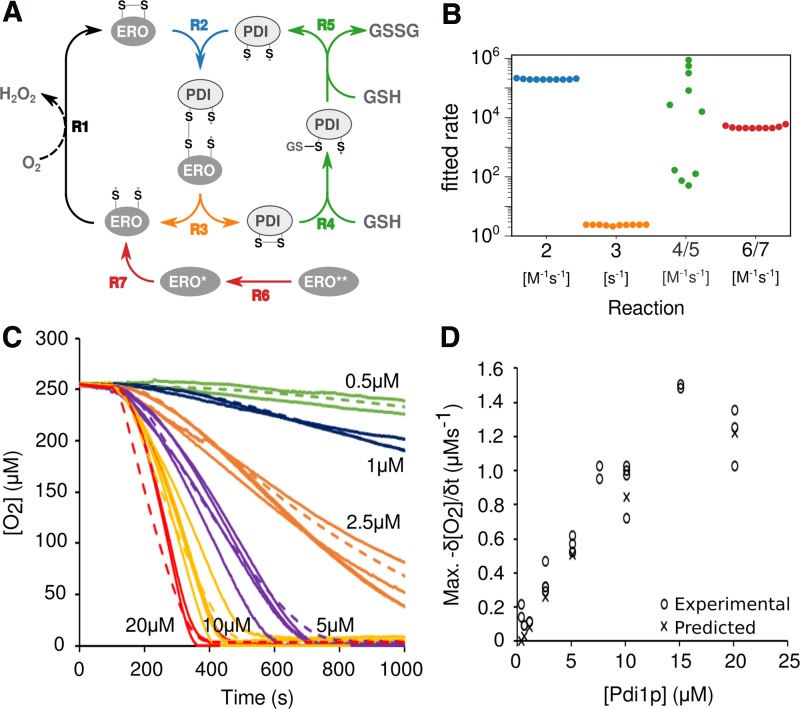
**Kinetic Modeling of GSH-promoted Pdi1p/Ero1p interaction. (A)** Representation of the model showing the reaction steps incorporated to define the electron transfer from GSH to molecular O_2_. **(B)** A comparison of 10 separate model repeats for each of the terms defined in **(A)**. R1 was determined experimentally and was, therefore, not incorporated in the fitting. **(C)** The bulk experimental O_2_ consumption data where the *solid lines* are *color coded* to show [Pdi1p] compared with the O_2_ consumption data generated by the best fitting data, *dashed lines*, that is, the best fitting data were those achieved using the parameters from the 3rd of the 10th modeling replicates shown in Figure 6B. **(D)** Comparison of the experimentally observed (○) maximum for δ[O_2_]/δt with that calculated using the data predicted ( × ) by the model. Color images are available online.

We constructed the final model based on these considerations (Fig. 7A) and implemented the model in the biochemical simulation software, Complex Pathway Simulator (COPASI) (21). After initiation of the model with the known input concentrations of the molecular components, we then sought to determine the reaction rates by parameter fitting to our experimentally determined O_2_ consumption in the system.

We initially observed that the model found well-fitting solutions with very different parameter combinations, indicating that the model was strongly underdetermined. We, therefore, experimentally determined rates for Ero1p oxidation, by observing O_2_ consumption in reactions containing only Ero1p and varying concentrations of DTT (Supplementary Fig. S4).

In addition, we characterized the diffusion of environmental O_2_ into the Clarke cell by depleting O_2_ with sodium dithionite, and by determining rates of recovery of the O_2_ concentration in the open cell (Supplementary Fig. S5).

When we fixed the rates for these reactions using the experimentally determined values and repeated the parameter fitting exercise, all rate constants except the Pdi1p:GSH interaction were reproducibly determined within very narrow ranges (Fig. 7B and Supplementary Fig. S6). When compared with the experimental O_2_ consumption data (○), the predicted O_2_ consumption curves ( × ) at different Pdi1p and Ero1p concentrations showed excellent correlation (Fig. 7C), with a nonlinear increase in maximum δ[O_2_]/δt when the Pdi1p concentration is increased >15:1 Pdi1p:Ero1p (Fig. 7D).

### *In vitro* rates and their relationship with OPF activity *in vivo*

The measuring of biochemical activity *in vitro*, and the interpretation of this activity *in silico*, provides quantitative information that can be used to explore characteristics of the OPF pathway *in vivo*.

Intracellular levels of Pdi1p and Ero1p in yeast have been reported as 28,000 and 5200 molecules per cell, respectively, in recent high-quality proteome data sets (22). Given estimated ER volumes of 0.2–0.4 μm^3^ (62), this would be equivalent to concentrations of 155 and 29 μ*M*. To estimate the substrate flux processed by these factors, we used recent proteomics studies (15, 23, 51, 60, 63) to identify all proteins residing in or passing through the ER (*i.e.*, all proteins reported to be present in the ER, Golgi, endosome, vacuole, cell wall, or reported to be extracellular).

We combined these data with published protein turnover rates (9) and with the number of cysteines per protein, to arrive at an estimate of around 269,000 cystines formed per minute. This assumes that every single cysteine pair present in all proteins passing through the ER is converted to a cystine through Pdi1p activity, which is unlikely to be the case. This value is thus an upper limit only, and likely substantially overestimates the need for catalyzed disulfide bond formation.

In our *in vitro* experiments, the maximal capacity of Pdi1p for disulfide bond formation (*i.e.*, when both Ero1p and GSH are provided in excess) is ∼15 disulfide bonds per minute per Pdi1p molecule (estimated from the data shown in Fig. 3D). Thus, if sufficient Ero1p is present *in vivo*, the estimated Pdi1p population in yeast could process up to 400,000 disulfide bonds per minute.

The *in vivo* Ero1:Pdi1 ratio of roughly 1 in 5 is slightly below the ratio where Ero1p becomes saturating *in vitro* (2:5, Fig. 3D), indicating that *in vivo* Pdi1p activity might be limited by Ero1p availability. In contrast, Pdi1p can also act as an isomerase in addition to forming disulfide bonds, in reactions that are net neutral in terms of electron flow but that nevertheless remove Pdi1p molecules from the active pool while the isomerization reactions are being catalyzed. This would reduce the effective Pdi1p concentration itself.

To analyze how well these estimates reflect the biology of yeast cells, we tested experimentally how such cells cope with an enforced reduction of their Pdi1p content, using strains in which the original promoter was replaced by a Tet-responsive tetO7promoter that is repressible by addition of doxycycline to the growth medium (6).

**FIG. 8.**
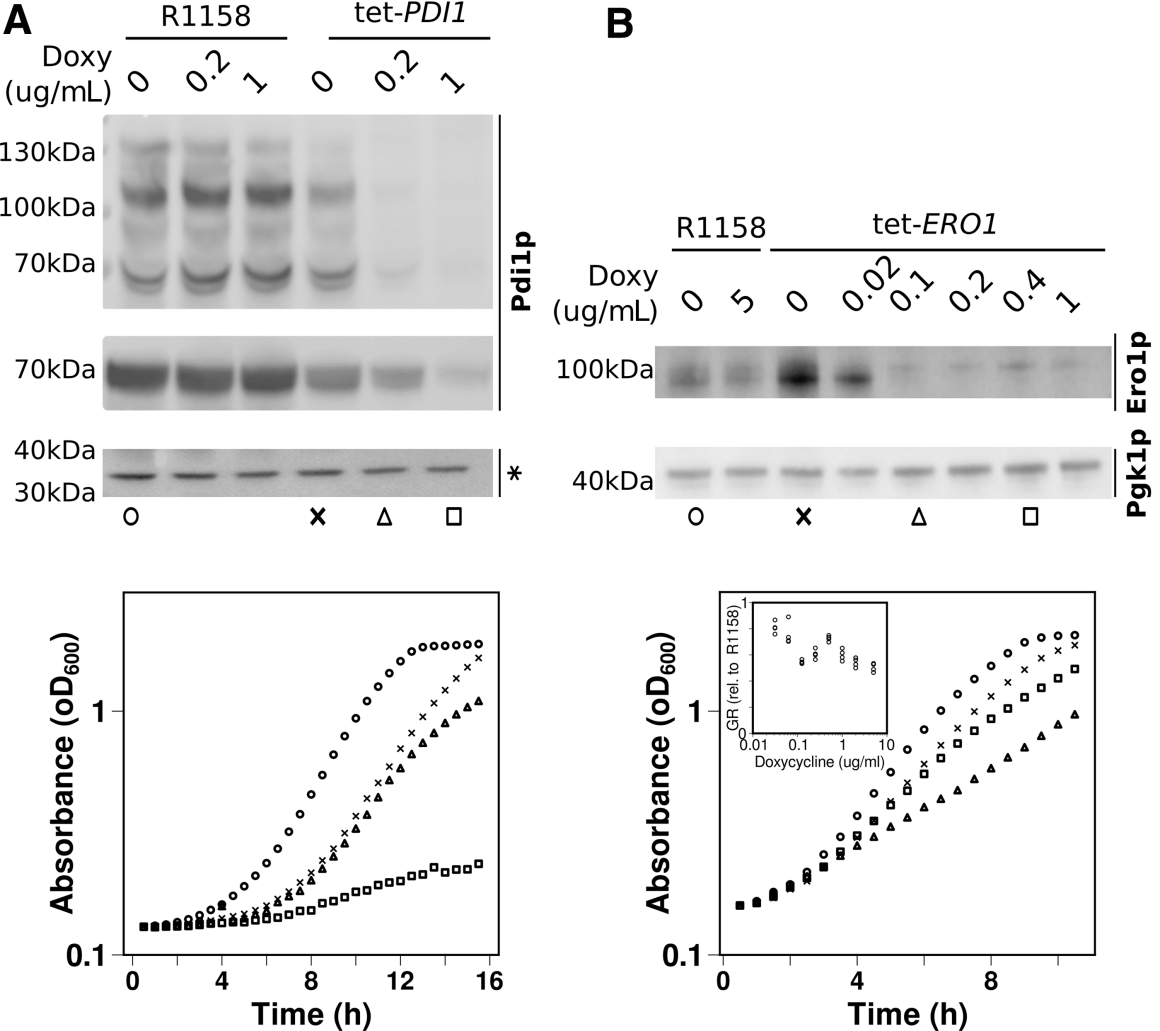
**Yeast fitness correlates with intracellular levels of Pdi1p and Ero1p.** This figure presents analyses of strains where expression of the *PDI1* or *ERO1* genes was placed under control of a doxycycline-repressible promoter (Tet-Pdi1 and Tet-Ero1, respectively), compared with isogenic control strains containing the natural promoters (R1158). **(A)** *Top panel:* anti-Pdi1 Western blots of samples treated with 5k PEG maleimide that shifts reduced Pdi1p to higher molecular weight, anti-Pdi1 Western blots of non-PEG maleimide-treated protein showing total Pdi1p content per cell, and loading controls for the Pdi1p Western blots (a lower molecular weight nonspecific band cross-reacting with the anti-Pdi1p antibody). *Bottom panel:* growth curves for selected strains and conditions, corresponding to symbols underneath the Western blots. **(B)** *Top panel:* anti-Ero1 Western blot of non-PEG maleimide-treated samples, and loading control (Pgk1p). *Bottom panel:* growth curves for selected strains and conditions, corresponding to symbols underneath the blots. The *inset graph* shows growth rates of Tet-*ERO1* strains, expressed relative to the growth rate of R1158, for a wider range of doxycycline concentrations. PEG, polyethylene glycol; Tet, tetracycline.

We observed that substantial Pdi1p depletion can be tolerated with only a minor reduction in growth rates. At the highest doxycycline concentration used in our experiment, Pdi1p concentrations were reduced to 23% of the wild-type content (Fig. 8A and Supplementary Fig. S7A), yet this still permitted growth at 24% of the wild-type growth rate. Moreover, the ratio of observable Pdi1p oxidation states was not substantially affected by this depletion either (Fig. 8B or Supplementary Fig. S7B).

In contrast to the monotonic decrease of both Pdi1 content and growth rates with doxycycline concentration in a tetracycline (Tet)-*PDI1* strain, Ero1p levels showed a more complex relationship between the corresponding parameters (Fig. 8B and Supplementary Fig. S7C). In the absence of doxycycline, the promoter replacement strain contained higher levels of Ero1p than the control. Interestingly, however, this increased expression coincided with a reduction in growth rates of 5%–10%. Upon addition of doxycycline at increasing concentrations, Ero1p levels initially dropped but then recovered slightly around the 0.4 mg/L mark. Growth rates moved parallel to the Ero1p content, initially dropping before recovering and then dropping again (cf the inset panel in Fig. 8B).

Interestingly, in our blot, the Ero1p band displayed as an apparent double band, and the depletion of Ero1p in the doxycycline-repressible strain coincided with a clear shift from a predominantly lower molecular weight isoform to a higher molecular weight isoform and low Ero1p levels. We interpret this overall complex behavior as evidence for some currently not understood regulation of Ero1p expression, possibly involving post-translational modifications that may be regulated by feedback loops (since it is dependent on intracellular Ero1p levels).

Notably though, like the Tet-*PDI1* strain, the Tet-*ERO1* strain allows substantial growth to occur even when this protein is depleted to <25% of wild-type levels. These observations corroborate our *in silico* prediction that the oxidative capacity in *S. cerevisiae in vivo* comfortably exceeds the required capacity.

## Discussion

*S. cerevisiae* is a well-exploited host system for the production of secreted recombinant proteins due, in part, to its potential to carry out post-translational modifications such as inter- and intramolecular disulfide bond formation. However, the secretory capacity of *S. cerevisiae* cells is far less than that of cultured mammalian cells, presumably as a consequence of a less developed OPF pathway that has not evolved to handle the secretion of large amounts of secretory proteins. Yet this yeast species has been successfully engineered to yield commercially viable levels, but this has been done empirically rather than in a directed and informed manner.

The latter requires a systematic understanding of the yeast OPF pathway and its regulation using both *in vivo* and *in vitro* approaches, with the latter being hampered by inefficient methods for isolating key OPF components, in particular the ER-located oxidase Ero1p (57).

In this study, we have developed an efficient reduction/oxidation processing strategy to generate homogenously oxidized yeast Ero1p expressed in *E. coli* strains. This new methodology has for the first time enabled the *in vitro* quantitative assessment of the interaction of Pdi1p and Ero1p by measuring O_2_ consumption and glutathione disulfide (GSSG) formation through nicotinamide adenine dinucleotide phosphate (NADPH)-coupled assays. The resulting quantitative data have allowed us to generate a simple model that can describe the oxidizing capacity of Pdi1p and Ero1p *in vitro* and predict the *in vivo* effect of modulation of the levels of the key components of the OPF, thereby allowing us to begin to explore yeast oxidative folding in a quantitative way. Overall, our study informs and provides new insights into how OPF works at a molecular level.

One fundamental difference between yeast and mammalian PDI and endoplasmic reticulum oxidase 1 (ERO1) proteins is that the yeast proteins are glycosylated *in vivo*, whereas here we carry out *in vitro* studies exclusively with nonglycosylated proteins produced in *E. coli*. The report that the specific activities of the natural glycosylated yeast Pdi1p versus a recombinant nonglycosylated form are similar (31) would suggest that the presence or absence of N-glycans has little or no effect on the isomerase activity of this enzyme, although detailed equivalent comparisons for Ero1p have not to our knowledge been reported in the literature.

Overall, our study informs and provides new insights into how OPF works at a molecular level. The yeast ER is more strongly oxidizing than the cytoplasm, with a GSH:GSSG ratio of 1:1 to 3:1 in the ER compared with 30:1 to 100:1 in the cytoplasm (24), meaning that GSH concentrations may reach 10 m*M* based on measured total GSH concentrations in the ER of HeLa cells (35). If GSH is readily available as an electron source for the oxidative folding machinery, it is not clear how proteins could compete for access to Pdi1p.

Our observations of the extreme pH dependence of Pdi1p oxidation by GSH, but not by a protein substrate (Fig. 5), suggest that this interference is not relevant at the reported pH within the ER (12). The potential for continuous futile cycles of GSH-mediated reduction and oxidation was proposed as a significant issue (57) to the “active site asymmetry” model of Pdi1p activity proposed by Kulp *et al.* (30). The observation of the strong pH dependence of such futile cycles, therefore, adds weight to this model.

Interestingly, pH dependence has also been observed for the human PDI/ERO interaction (4). Assessment of the oxidation of rdRNase A in the absence of either Pdi1p or Ero1p, at pH 8 where spontaneous oxidation would be faster than in the ER where the pH is 7.5, shows that nonenzyme-mediated substrate oxidation is far slower than that observed in the catalyzed processes (Supplementary Fig. S8) and, therefore, are not able to account for difference in activity between the two electron sources.

Another observation that is consistent with the “active site asymmetry” model, which posits that one active site is utilized as an oxidase and the other as a reductase and an isomerase, is our inability to observe completely oxidized Pdi1p, either *in vitro* or *in vivo*. At the end of the *in vitro* experiment, when reduced RNase A is consumed, the resting state of the Pdi1p contains a significant proportion of Pdi1p containing a single reduced disulfide (Fig. 6A, B and Supplementary Figs. S2 and S3). Similarly, even when Pdi1p levels are significantly reduced *in vivo*, the proportion of reduced oxidized protein is not altered (Fig. 8A and Supplementary Fig. S7B).

Comparison of the modeled system with the *in vivo* environment of the yeast ER suggests that the total oxidation capacity of a yeast cell is 400,000 disulfide bonds per minute, not considering alternative roles of Pdi1p such as isomerization, which would diminish this capacity. *In silico* assessment of the secretory pathway, which OPF is part of, suggests a maximum requirement of 269,000 disulfides to be formed per minute. This is likely a substantial over estimate of the real required rate, since few proteins contain all their cysteines within Pdi1p-catalyzed disulfide bonds. Depletion experiments confirm the notion that Pdi1p is provided in substantial excess over requirements of wild-type strains.

**FIG. 9.**
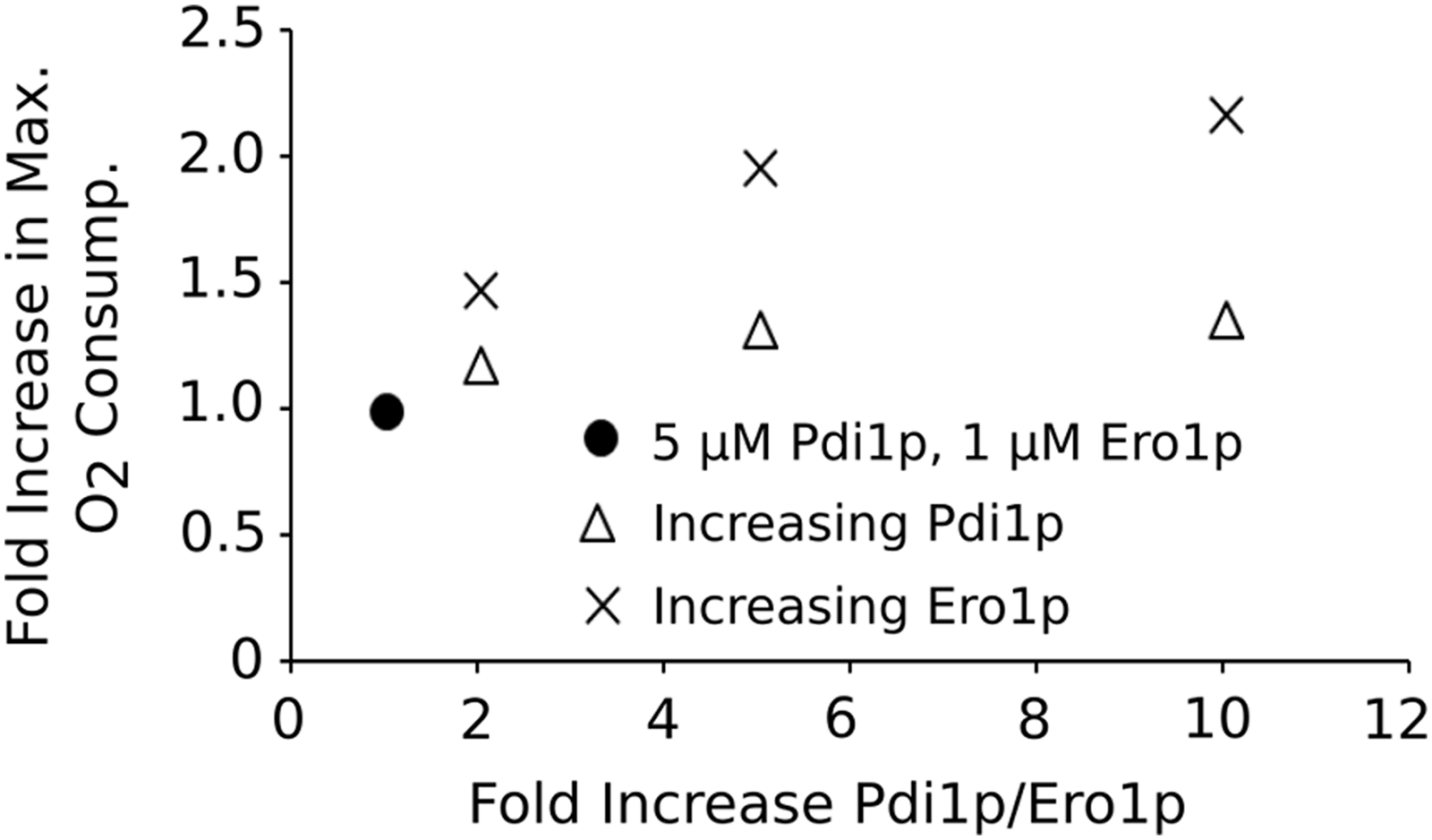
**Predicted increase in oxidative folding capacity on Pdi1p/Ero1p overexpression**. The increase in oxidative folding capacity of the Pdi1p/Ero1p system when the concentration of the OPF components is increased. ●, 5 μ*M* Pdi1p and 1 μ*M* Ero1p; × , increase in Ero1.

These observations appear at odds with the fact that Pdi1p overexpression can enhance secretion of a variety of recombinant proteins (14, 47). Our quantitative model predicts that 10-fold elevation in the steady-state levels of Pdi1p should only increase the capacity of the OPF pathway by 1.4-fold, whereas similar overexpression of Ero1p should more than double the capacity of the pathway (Fig. 9). Thus, manipulating Ero1p levels might provide a more promising strategy compared with manipulating Pdi1p levels.

A number of studies have indeed shown that Ero1p overexpression can improve production of a range of recombinant substrates, from single chain variable fragments (scFvs) to T cell receptors, and for some substrates including different scFvs, Ero1p overexpression was indeed more beneficial than Pdi1p overexpression (61). Similarly, de Ruijter *et al.* (45) showed that increasing levels of Ero1p consistently enhanced titers of an antibody product expressed in yeast more than was the case when Pdi1p levels were increased (45). However, these studies also demonstrate significant variability and substrate specificity in the behavior of different recombinant products.

It is likely that target recombinant mammalian proteins will have evolved in the significantly more complex environment of the mammalian OPF pathway and may interact suboptimally with yeast Pdi1p. This may explain why in some cases, individual substrates respond well to Pdi1p overexpression.

In conclusion, we have developed a novel method for the processing of Ero1p that enables, for the first time, an understanding of the *in vitro* concentration dependencies of the Pdi1p/Ero1p catalytic cycle. Based on a suite of biochemical assays, we generated a detailed computational model that accurately describes the interaction of Pdi1p and Ero1p for the oxidation of substrates. This model gives new insights into the yeast OPF and should form a platform on which to design future engineering strategies to enhance the secretion of authentic high-value recombinant proteins from *S. cerevisiae.*

## Materials and Methods

### Plasmid construction

The plasmid encoding a codon optimized version of Ero1p_19-424_ was a kind gift from Lloyd Ruddock (University of Oulu). The plasmid expressing yeast Pdi1p_22-530_ without its N-terminal signal sequence was produced by polymerase chain reaction amplification of the relevant *PDI1* sequence from plasmid pUKC470 using a forward primer (GCGATGCATCACCATCACCACCATATGCAACAAGAGGCTGTGGCC) and a reverse primer (CGCGCGGGATCCCGCTATTACAATTCATCGTGAATGGCATCTTCTTC) followed by digestion using *Nsi*I and *Bam*HI (Promega, Madison, Wisconsin) and ligation into a pET15b vector.

### Protein production

Pdi1p- and Ero1p-encoding plasmids were transformed into the BL21 [DE3] pLysS strain of *E. coli* (Invitrogen, Carlsbad, CA) by heat shock at 42°C for 60 s. Transformants were selected by growth on LB (Lysogeny broth; 1% w/v tryptone, 0.5% w/v yeast extract, 1% w/v NaCl) agar plates containing 35 μg/mL chloramphenicol (Sigma Aldrich, St Louis, MO) and 100 μg/mL ampicillin (Sigma Aldrich). Individual transformed colonies were used to inoculate 10 mL of LB containing 35 μg/mL chloramphenicol and 100 μg/mL ampicillin and then grown overnight at 37°C.

The resulting cultures were centrifuged at 1359 × *g*, the supernatant was removed, and the resulting cell pellet was resuspended in 1 mL of LB and then used to inoculate 1 L of LB containing 35 μg/mL chloramphenicol and 100 μg/mL ampicillin. The cultures were grown to an OD_600_ of 0.6 before gene expression was induced as follows. Pdi1p synthesis was induced by 1 m*M* isopropyl β-d-thiogalactopyranoside (IPTG; Melford Laboratories, Ipswich, Suffolk) and incubated for 4 h at 30°C.

Ero1p synthesis was induced by addition of 1 m*M* IPTG and 10 μ*M* FAD (Sigma Aldrich) followed by incubation at 20°C for 12–16 h. The cells were harvested by centrifugation at 1359 × *g* for 10 min before resuspension in 20 m*M* phosphate, 50 m*M* sodium chloride (NaCl), pH 7.4, plus protease inhibitor cocktail (Sigma Aldrich).

### Protein purification: Ero1p

Cells producing Ero1p were disrupted by sonication and the resulting lysate was treated with 2 *M* magnesium chloride (MgCl_2_, 10 m*M* final concentration) and deoxyribonuclease A (DNase A, 150 U/L culture; Sigma Aldrich) and incubated for 30 min at room temperature.

The resulting lysate was clarified by centrifugation at 27,216 *g* for 30 min at 4°C. The supernatant was removed and treated with 5 *M* NaCl and 1 *M* imidazole to give 0.5 *M* and 10 m*M* concentrations, respectively, before incubation with Ni^2+^ loaded nitrilotriacetic acid modified sepharose (Ni-NTA) resin formed from chelating sepharose (2 mL of resin per 1 L of culture purified; GE Healthcare, Chicago, IL) for 1 h at room temperature. The resin was collected and washed with 10 mL of 20 m*M* phosphate, 50 m*M* NaCl, 10 m*M* imidazole, pH 7.4, and then 10 mL of 20 m*M* phosphate, 50 m*M* NaCl, pH 7.4, before elution of bound proteins with 1 mL fractions of 20 m*M* phosphate, 50 m*M* NaCl, 50 m*M* ethylenediaminetetraacetic acid (EDTA), pH 7.4.

The resulting solution was diluted to 100 μ*M* protein concentration before being treated with 30 m*M* DTT (Melford Laboratories) and incubated at room temperature for 1 h. Any precipitated material was removed by centrifugation at 20422 × *g* for 10 min before DTT removal by buffer exchange with PD10 cartridges (GE Healthcare) equilibrated with 20 m*M* phosphate, 50 m*M* NaCl, pH 7.4. The approximate protein concentration was determined by absorbance at 280 nm before a 10-fold molar excess of FAD (∼750 μ*M*) was added to the mixture and incubated for 12–16 h at room temperature.

The mixture was concentrated using a 10 kDa molecular weight cutoff (MWCO; Pall, NY) centrifugal filter before loading onto a Superdex 200 16/600 gel column (GE Healthcare) equilibrated with 20 m*M* phosphate, 50 m*M* NaCl, pH 7.4. The appropriate fractions were collected, protein concentration was determined in the presence of 0.1% sodium dodecyl sulfate to determine protein:FAD ratio, aliquoted, and stored at −80°C.

### Protein purification: Pdi1p

The Pdi1p purification protocol was based on that described by Lappi and Ruddock (32). In brief, cells producing Pdi1p were lysed by incubation at room temperature in the presence of 0.5% (v/v) Triton X-100 and a combination of DNase A (150 U/L culture) and MgCl_2_ (10 m*M*) for 30 min.

The resulting lysate was clarified by centrifugation at 20422 × *g* for 30 min at 4°C and then incubated with Ni-NTA (1 mL/L culture) for 2 h at room temperature. The resin was then collected and washed with 10 mL of 20 m*M* phosphate, 50 m*M* NaCl, 10 m*M* imidazole, pH 7.4 and then 10 mL of 20 m*M* phosphate, and 50 m*M* NaCl, pH 7.4. The bound protein was eluted using 1 mL fractions of 20 m*M* phosphate, 50 m*M* NaCl, and 50 m*M* EDTA, pH 7.4, and then buffer exchanged into 20 m*M* phosphate and 50 m*M* NaCl, pH 7.4, using a PD10 column.

The resulting mixture was purified using a 5 mL HiTrap Q FF anion exchange column (GE Healthcare) with a nonlinear gradient between 20 m*M* phosphate, 50 m*M* NaCl, pH 7.4 (buffer A), and 20 m*M* phosphate, 1 *M* NaCl, pH 7.4 (buffer B) (postinjection 12 mL buffer A, 2 mL of 0%–20% buffer B, 20 mL of 20%–75% buffer B, 15 mL of 75%–100% buffer B, and 10 mL of 100% buffer B). The appropriate fractions were then combined and concentrated using a 10 kDa MWCO centrifugal filter before aliquoting at freezing at −20°C.

### Preparation of rdRNase A

Bovine pancreatic RNase A (Sigma Aldrich) was dissolved in 20 m*M* phosphate, 8 *M* urea, pH 8, to a concentration of ∼750 μ*M* and then treated with DTT at a final concentration of 30 m*M*. The reaction mixture was incubated for 4 h at room temperature before quenching the reaction by lowering the pH to 3 with acetic acid (AcOH). The buffer was then exchanged to 0.1 *M* AcOH using PD10 columns. The protein concentration was determined and samples were aliquoted before lyophilization to dryness.

### O_2_ consumption assay

O_2_ consumption was measured using a Clarke O_2_ electrode (DM10; Rank Brothers, Bottisham, Cambridge) in conjunction with a Pico Technologies Data Logger and PicoLog software (Pico Technologies, St Neots, Cambridgeshire). A 1 mL chamber was utilized and a final reaction volume of 600 μL was used for each experiment.

### GSH-mediated O_2_ consumption assay

Stock solutions of GSH (Sigma Aldrich) were prepared fresh before the start of every experiment and stored on ice. The thiol concentration of these stock solutions was calculated using Ellman's assay (11) and this concentration was used to determine the volume to be added to give 40 m*M* GSH in the electrochemical cell.

Purified Pdi1p was combined with 10 × O_2_ consumption buffer (1 *M* tris-acetic acid buffer [Tris-HAc], 500 m*M* NaCl, pH 8), H_2_O, and GSH (40 m*M*), and the baseline O_2_ level was allowed to stabilize for 100 s before addition of Ero1p to the electrochemical cell.

### DTT-mediated O_2_ consumption assay

Purified Ero1p (1 μ*M*) was combined with 10 × O_2_ consumption buffer and H_2_O and the baseline O_2_ level was allowed to stabilize for 100 s before addition of 1 *M* DTT.

### Analysis of RNase A oxidation by AMS trapping

Lyophilized rdRNase A was dissolved in 0.1 *M* AcOH to 765 μ*M*. Purified Pdi1p, 10 × O_2_ consumption buffer, H_2_O, and RNase A (60 μ*M*) (Sigma Aldrich) were combined and the baseline O_2_ level was allowed to stabilize for 100 s before addition of purified Ero1p (variable μ*M*).

Over the duration of the experiment, 10 μL aliquots were removed from the electrochemical cell and quenched by the addition of 10 m*M* of 4-acetamido-4′-maleimidylstilbene-2,2′-disulfonic acid (AMS; Molecular Probes, Invitrogen, Carlsbad, CA) to give a final concentration of 1 m*M*.

The samples were incubated for 1 h before treatment with 4 × sodium dodecyl sulfate–polyacrylamide gel electrophoresis (SDS-PAGE) loading dye +5% (v/v) 2-mercaptoethanol (Sigma Aldrich) and then heated to 95°C for 5 min.

Three microliters of each sample was run on a 15% tris-glycine buffer (Tris-Gly) SDS-PAGE at 180 V and then stained with Instant Blue (Expedeon, Over, Cambridge) stain. The resulting gels were scanned using an Epson Perfection 3200 flatbed scanner and band analysis was carried out using ImageJ software (46).

### *In vitro* analysis of Pdi1p oxidation state by PEG trapping

Six hundred microliters O_2_ consumption assays as already described, with rdRNase A as an electron source and varying Pdi1p and Ero1p concentrations, were analyzed. At different time points, 10 μL samples were removed from the electrode chamber and trapped by the addition of 1 μL of 200 m*M* 5k PEG maleimide solution (Sigma Aldrich). The sample was thoroughly mixed and incubated for at least 30 min before the addition of 4 × SDS-PAGE loading buffer incorporating 5% (v/v) 2-mercaptoethanol. The samples were then boiled at 95°C and analyzed using SDS-PAGE and Western blot. Polyclonal antibodies were raised in a rabbit against purified Pdi1p and were produced by Capra Science (Ängelholm, Sweden).

### *In vivo* analysis of Pdi1p oxidation state by PEG trapping

To allow for regulation of the expression of the *PDI1* gene, the tet-O promoter was introduced into the yeast strain R1158 in place of the native *PDI1* gene promoter. This strain enabled knockdown of *PDI1* expression and was constructed using the KanMX4-tetO cassette method developed by Yen *et al.* (64). The resulting strain was named tet-*pdi1*.

Overnight cultures of R1158 and tet-*pdi1* were reinoculated to an OD_600_ of 0.2 in fresh yeast extract peptone dextrose (2% w/v glucose, 1% w/v yeast extract, and 2% w/v bactopeptone) and grown for 6 h in the absence or presence of doxycycline (Sigma Aldrich) and added to a final concentration of either 0.02 μg/mL (45 n*M*) or 0.1 μg/mL (225 n*M*).

After 6 h, five OD_600_ units of cells were harvested at 16,100 *g* and proteins were extracted in cell lysis buffer (1 × phosphate-buffered saline, 100 m*M* NaCl, 100 m*M* EDTA, pH 8.0, and 1 × protease inhibitor cocktail tablet) with or without 20 m*M* of 5k PEG maleimide using glass bead lysis. Proteins were analyzed using reducing SDS-PAGE and Western blot analysis. Polyclonal antibodies were raised in a rabbit against purified Pdi1p and were produced by Capra Science (Ängelholm, Sweden).

### Computational modeling of O_2_ consumption

A structural biology markup language version of the model file is available from the Biomodels database (17) under accession number MODEL1902080001. The fitting of experimental data to reactions was achieved using the COPASI 4.18 parameter estimation module (21). The average O_2_ consumption data were calculated for each Pdi1p and Ero1p concentration variable with the 100 s of baseline stabilization time removed. Each change in parameter was accompanied by 10 repeats of the fitting to determine reproducibility of the fits in terms of the derived k value.

To compare between each run, the average root-mean-squared-deviation (RMSD) for each experiment was calculated by averaging the values for each of the individual protein concentration variables obtained in each run. The rank order of fitting was defined by the lowest to highest RMSD value.

### Estimation of protein oxidation rates in yeast *in vivo*

The main data source to identify proteins passing through the ER was Wiederhold *et al.* (63), who reviewed and summarized information on mass spectrometry-based subcellular localization experiments from 18 different pre-2010 studies. A number of later studies (15, 23, 51, 60) were used to complete this set.

Abundance data for each protein were used as reported in Ho *et al.* (20), protein half-life data were from Christiano *et al.* (9), and a doubling time was assumed as 120 min. Abundance, decay-rate, and number of cysteines per protein are listed in Supplementary Table S1. The maximum number of disulfide bonds to be formed per minute was calculated as

\begin{align*}
 { k_ { Cys } } = \sum \nolimits_ { i = 1 } ^n { { A_i } \, \, \cdot \, \, \left\lfloor { \frac { { Cy { s_i } } }  { 2 } } \right\rfloor } \, \, \cdot \, \, \left( { { k_i } + { \frac { ln ( 2 ) }  { { t_d } } } } \right) ,
\end{align*}

where *A_i_* is the abundance of the *i*th protein and *Cys_i_* is the number of cysteines it contains, *k_i_* its degradation rate, and *t_d_* the doubling time of the growing culture. The equation is based on the standard math for first-order reactions, assuming that in the steady state, proteins need to be formed with a rate that balances their decay and their dilution through growth. It is assumed that only even number of cysteines can be oxidized.

## Supplementary Material

Supplemental data

Supplemental data

Supplemental data

Supplemental data

Supplemental data

Supplemental data

Supplemental data

Supplemental data

Supplemental data

## Abbreviations Used

AcOH: acetic acid
AMS: 4-acetamido-4′-maleimidylstilbene-2,2′-disulfonic acid
COPASI: Complex Pathway Simulator
DNase A: deoxyribonuclease A
DTT: 1,4-dithiothreitol
EDTA: ethylenediaminetetraacetic acid
ER: endoplasmic reticulum
ERO1: endoplasmic reticulum oxidase 1
Ero1p: purified yeast ERO1
FAD: flavin adenine dinucleotide
GSH: glutathione
GSSG: glutathione disulfide
H_2_O_2_: hydrogen peroxide
IPTG: isopropyl β-d-thiogalactopyranoside
LB: Lysogeny broth
MgCl_2_: magnesium chloride
MWCO: molecular weight cutoff
NaCl: sodium chloride
Ni-NTA: Ni^2+^ loaded nitrilotriacetic acid modified sepharose
O_2_: molecular oxygen
OPF: oxidative protein folding
PDI: protein disulfide isomerase
Pdi1p: purified yeast PDI
PEG: polyethylene glycol
rdRNase A: reduced and denatured ribonuclease A
RMSD: root-mean-squared-deviation
RNase A: ribonuclease A
scFv: single chain variable fragment
SDS-PAGE: sodium dodecyl sulfate–polyacrylamide gel electrophoresis
Tet: tetracycline
Tris-Gly: tris-glycine buffer
