# Supplementary material for: Quantitative Analyses of the Yeast Oxidative Protein Folding Pathway In Vitro and In Vivo
Source: Antioxid Redox Signal. 2019 Jun 24;31(4):261–74. doi: 10.1089/ars.2018.7615 (PMC6602113; doi:10.1089/ars.2018.7615)
Supplement: Supplemental data [file Supp_Fig1.pdf]

Supplementary Data

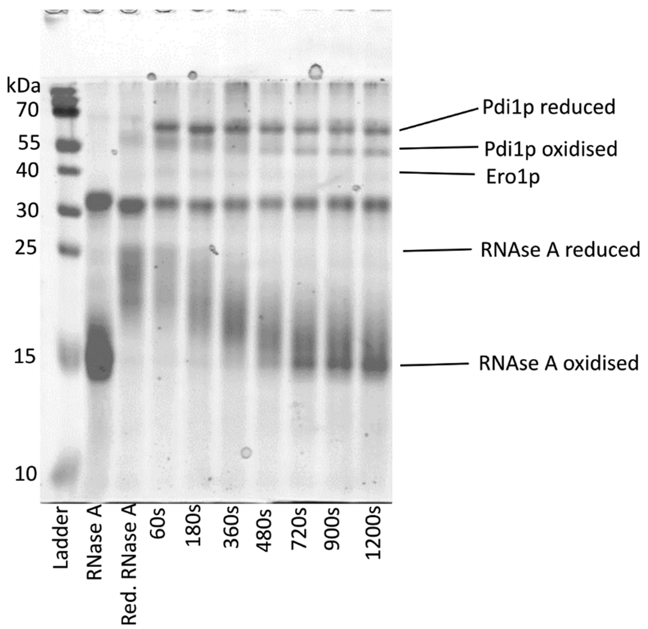

SUPPLEMENTARY FIG. S1. Full gel image of the data shown in Figure 4C. RNase A reoxidation over time catalyzed by 5 μM Pdi1p and 1 μM Ero1p was analyzed by reducing SDS-PAGE after AMS trapping. Pdi1p, Ero1p, and RNase A are marked on the gel. AMS, 4-acetamido-4′-maleimidylstilbene-2,2′-disulfonic acid; Ero1p, purified yeast endoplasmic reticulum oxidase 1; Pdi1p, purified yeast protein disulfide isomerase; RNase A, ribonuclease A; SDS-PAGE, sodium dodecyl sulfate–polyacrylamide gel electrophoresis.
