# Supplementary material for: Quantitative Analyses of the Yeast Oxidative Protein Folding Pathway In Vitro and In Vivo
Source: Antioxid Redox Signal. 2019 Jun 24;31(4):261–74. doi: 10.1089/ars.2018.7615 (PMC6602113; doi:10.1089/ars.2018.7615)
Supplement: Supplemental data [file Supp_Fig2.pdf]

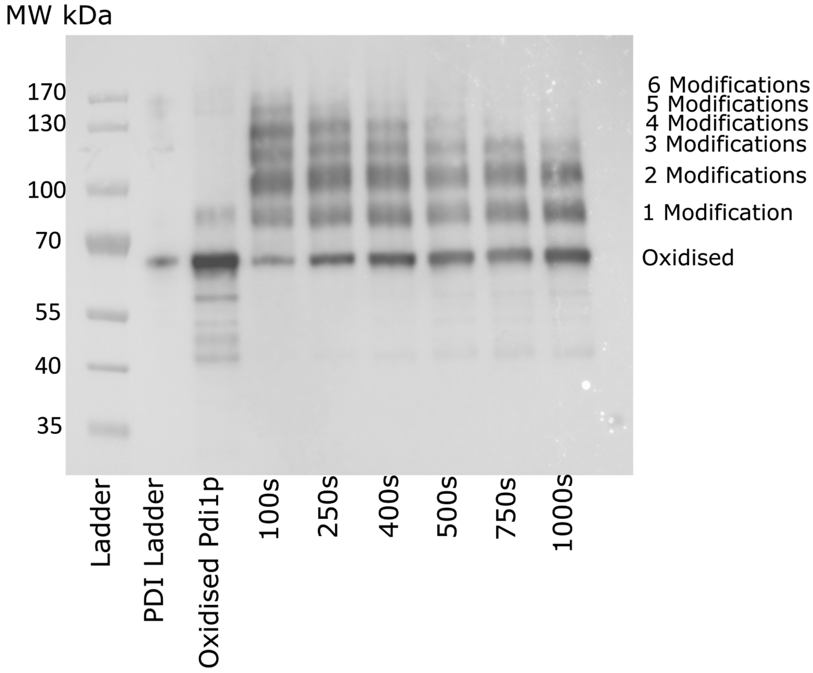

SUPPLEMENTARY FIG. S2. Full gel image of the data shown in Figure 6A. Pdi1p oxidation state over the course of RNase A reoxidation by Pdi1p (5 μM) and Ero1p (1 μM) was assessed by 5k PEG maleimide modification and SDS-PAGE/ Western blotting. PEG, polyethylene glycol.
