# Supplementary material for: Quantitative Analyses of the Yeast Oxidative Protein Folding Pathway In Vitro and In Vivo
Source: Antioxid Redox Signal. 2019 Jun 24;31(4):261–74. doi: 10.1089/ars.2018.7615 (PMC6602113; doi:10.1089/ars.2018.7615)
Supplement: Supplemental data [file Supp_Fig4.pdf]

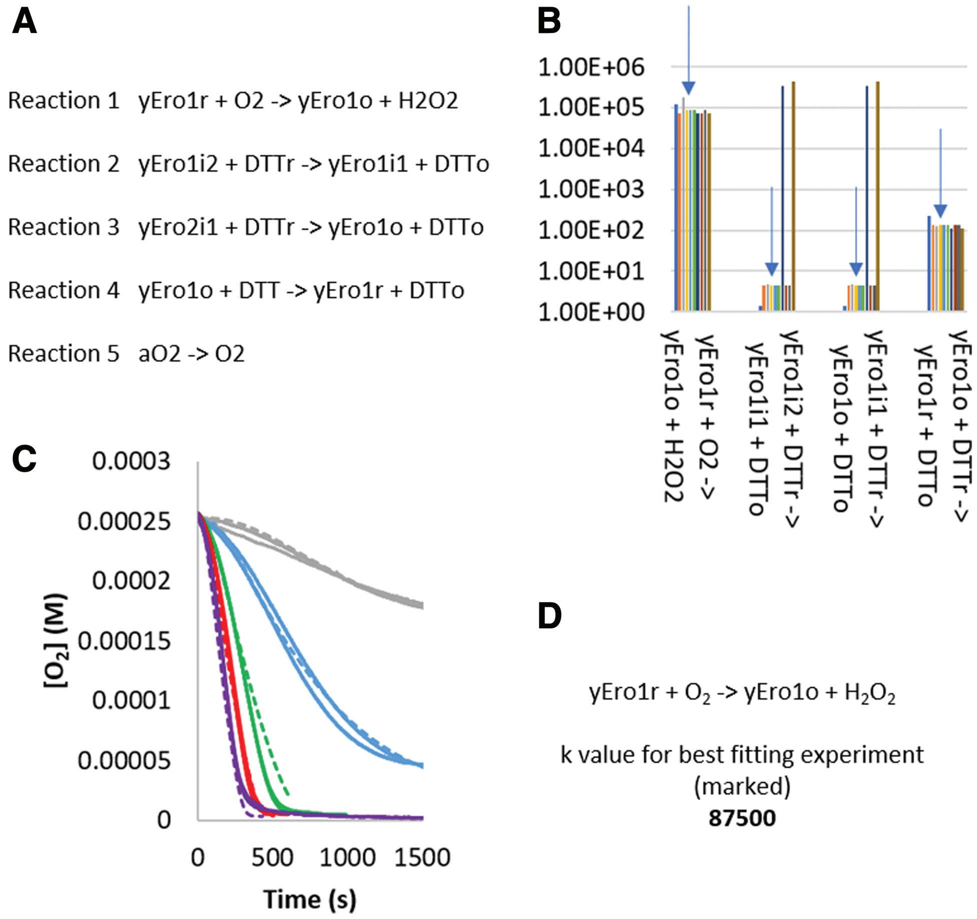

SUPPLEMENTARY FIG. S4. Determination of yEro1r oxidation rate constant from DTT reduction data. (A) Reaction schemes describing yEro1 reduction by DTT and reoxidation by O_2. Making the rate of Reactions 2 and 3 the same and that of Reaction 4 different gives a better fit for the data but also mimics the full model (Fig. 8A) better. **(B)** Parameter fitting of O_2 consumption data from Ero1p reduction with differing concentrations of DTT was repeated 10 times, which gave excellent reproducibility. The run, marked with an *arrow*, gave the best fitting data. **(C)** O_2 consumption plot of experimentally derived data, *solid lines*, plotted against the data predicted (*dashed lines*) using the rate terms determined by the best fitting run, marked with *arrows* on **(B)**. **(D)** The rate value for the reaction common to both the DTT reaction scheme and the full model. DTT, 1,4-dithiothreitol.
