# Supplementary material for: Quantitative Analyses of the Yeast Oxidative Protein Folding Pathway In Vitro and In Vivo
Source: Antioxid Redox Signal. 2019 Jun 24;31(4):261–74. doi: 10.1089/ars.2018.7615 (PMC6602113; doi:10.1089/ars.2018.7615)
Supplement: Supplemental data [file Supp_Fig5.pdf]

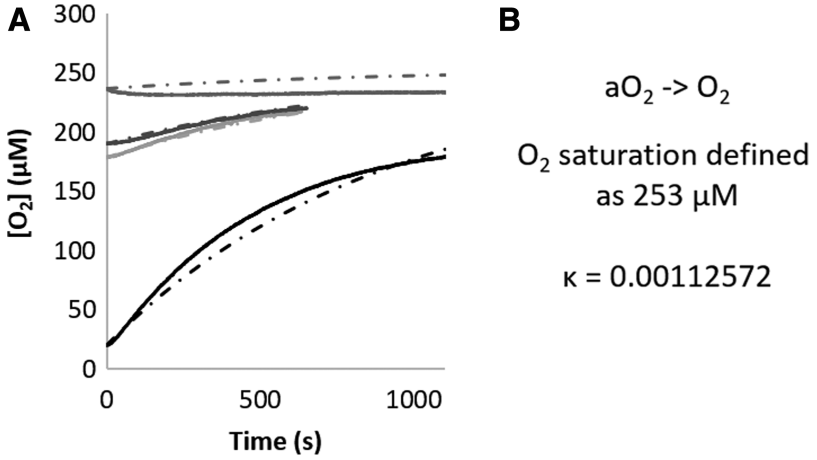

SUPPLEMENTARY FIG. S5. Determination of the rate of O_2 diffusion into the electrochemical cell during experiments. (A) *Solid lines:* O_2 diffusion back into the electrochemical cell after sodium dithionite depletion of O_2. *Dotted lines:* COPASI modeled fit of O_2 diffusion into electrochemical cell. **(B)** Reaction term used to define the O_2 diffusion into the electrochemical cell. The rate term, k, used in the full model. aO_2, atmospheric oxygen; COPASI, Complex Pathway Simulator; O_2, molecular oxygen.
