# Supplementary material for: Quantitative Analyses of the Yeast Oxidative Protein Folding Pathway In Vitro and In Vivo
Source: Antioxid Redox Signal. 2019 Jun 24;31(4):261–74. doi: 10.1089/ars.2018.7615 (PMC6602113; doi:10.1089/ars.2018.7615)
Supplement: Supplemental data [file Supp_Fig6.pdf]

$$\frac{\delta([H_2O_2] * V_{Compartment})}{\delta t} = +V_{Compartment} * (k1 * [yEro1r] * [O_2])$$

$$\frac{\delta([O_2] * V_{Compartment})}{\delta t} = -V_{Compartment} * (k1 * [yEro1r] * [O_2]) + V_{Compartment} * (k8 * (0.000253 - [O_2]))$$

$$\frac{\delta([yEro1o] * V_{Compartment})}{\delta t} = +V_{Compartment} * (k1 * [yEro1r] * [O_2]) - V_{Compartment} * (k2 * [yEro1o] * [yPDIr]) + V_{Compartment} * (k7 * [yEro1i] * [yPDIr])$$

$$\frac{\delta([yEro1r] * V_{Compartment})}{\delta t} = -V_{Compartment} * (k1 * [yEro1r] * [O_2]) + V_{Compartment} * (k3 * [yEro1yPDI])$$

$$\frac{\delta([yEro1yPDI] * V_{Compartment})}{\delta t} = +V_{Compartment} * (k2 * [yEro1o] * [yPDIr]) - V_{Compartment} * (k3 * [yEro1yPDI])$$

$$\frac{\delta([yPDIr] * V_{Compartment})}{\delta t} = -V_{Compartment} * (k2 * [yEro1o] * [yPDIr]) + V_{Compartment} * (k5 * [yPDIGSH] * [GSH]) - V_{Compartment} * (k6 * [yEro1i2] * [yPDIr]) - V_{Compartment} * (k7 * [yEro1i1] * [yPDIr])$$

$$\frac{\delta([yPDIo] * V_{Compartment})}{\delta t} = +V_{Compartment} * (k3 * [yEro1yPDI]) - V_{Compartment} * (k4 * [yPDIo] * [GSH]) + V_{Compartment} * (k6 * [yEro1i2] * [yPDIr]) + V_{Compartment} * (k7 * [yEro1i1] * [yPDIr])$$

$$\frac{\delta([GSH] * V_{Compartment})}{\delta t} = -V_{Compartment} * (k4 * [yPDIo] * [GSH]) - V_{Compartment} * (k5 * [yPDIGSH] * [GSH])$$

**SUPPLEMENTARY FIG. S6. Differential equations describing Pdi1p and Ero1p-mediated $O_2$ consumption produced by COPASI.**

$$\frac{\delta([yPDIGSH] * V_{Compartment})}{\delta t} = +V_{Compartment} * (k4 * [yPDIo] * [GSH]) - V_{Compartment} * (k5 * [yPDIGSH] * [GSH])$$

$$\frac{\delta([GSSG] * V_{Compartment})}{\delta t} = +V_{Compartment} * (k5 * [yPDIGSH] * [GSH])$$

$$\frac{\delta([aO_2] * V_{Compartment})}{\delta t} = -V_{Compartment} * (k8 * (0.000253 - [O_2]))$$

$$\frac{\delta([yEro1i1] * V_{Compartment})}{\delta t} = +V_{Compartment} * (k6 * [yEro1i2] * [yPDIr]) - V_{Compartment} * (k7 * [yEro1i1] * [yPDIr])$$

$$\frac{\delta([yEro1i2] * V_{Compartment})}{\delta t} = -V_{Compartment} * (k6 * [yEro1i2] * [yPDIr])$$

| | | |
|---|---|---|
| **yEro1r + O2 -> yEro1o + H2O2** | **κ1** | **87500 $M^{-1}s^{-1}$** |
| **yEro1o + yPDIr -> yEro1yPDI** | **κ2** | **207234 $M^{-1}s^{-1}$** |
| **yEro1yPDI -> yPDIo + yEro1r** | **κ3** | **2.27739 $s^{-1}$** |
| **yPDIo + GSH -> yPDIGSH** | **κ4** | **73.9823 $M^{-1}s^{-1}$** |
| **yPDIGSH + GSH -> yPDIr + GSSG** | **κ5** | **73.9823 $M^{-1}s^{-1}$** |
| **yEro1i2 + yPDIr -> yEro1i1 + yPDIo** | **κ6** | **5327.7 $M^{-1}s^{-1}$** |
| **yEro1i1 + yPDIr -> yEro1o + yPDIo** | **κ7** | **5327.7 $M^{-1}s^{-1}$** |
| **aO2 -> O2** | **κ8** | **0.00112572 $s^{-1}$** |

**SUPPLEMENTARY FIG. S6.** (*Continued*)
