# Supplementary material for: Quantitative Analyses of the Yeast Oxidative Protein Folding Pathway In Vitro and In Vivo
Source: Antioxid Redox Signal. 2019 Jun 24;31(4):261–74. doi: 10.1089/ars.2018.7615 (PMC6602113; doi:10.1089/ars.2018.7615)
Supplement: Supplemental data [file Supp_Fig7.pdf]

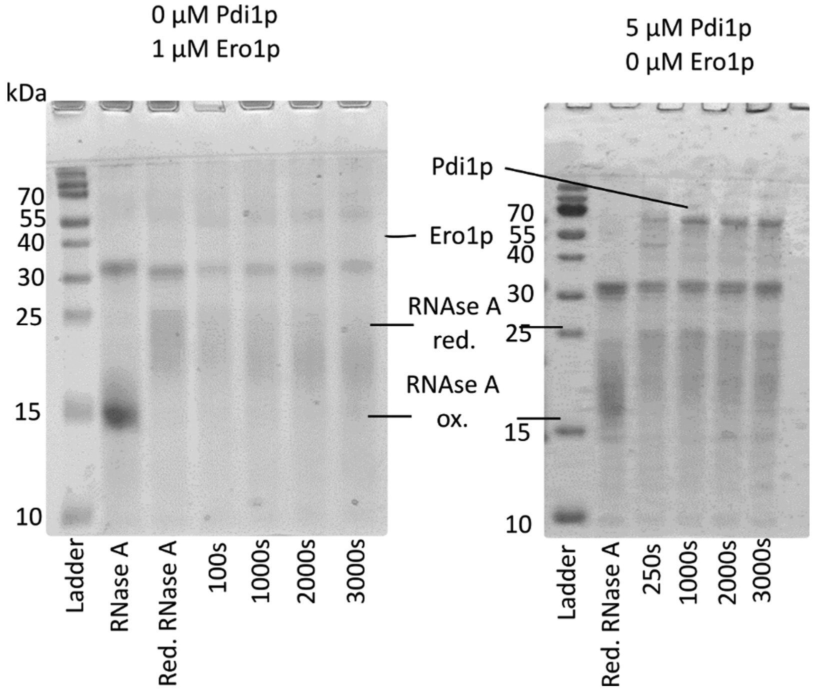

SUPPLEMENTARY FIG. S7. Full gel image of the data shown in Figure 8. This figure presents analyses of strains where expression of *PDI1* or *ERO1* genes was placed under control of a doxycycline-repressible promoter (Tet-Pdi1 and Tet-Ero1, respectively), compared with isogenic control strains containing the natural promoters (R1158). **(A)** Levels of Pdi1p in the tet-*PDI1* strain without PEG maleimide trapping. **(B)** The redox state of Pdi1p by PEG maleimide trapping of the tet-*PDI1* strain. **(C)** The levels of Ero1p in the tet-*ERO1* strain. ERO1, endoplasmic reticulum oxidase 1; PDI, protein disulfide isomerase.
