# Supplementary material for: Quantitative Analyses of the Yeast Oxidative Protein Folding Pathway In Vitro and In Vivo
Source: Antioxid Redox Signal. 2019 Jun 24;31(4):261–74. doi: 10.1089/ars.2018.7615 (PMC6602113; doi:10.1089/ars.2018.7615)
Supplement: Supplemental data [file Supp_Fig8.pdf]

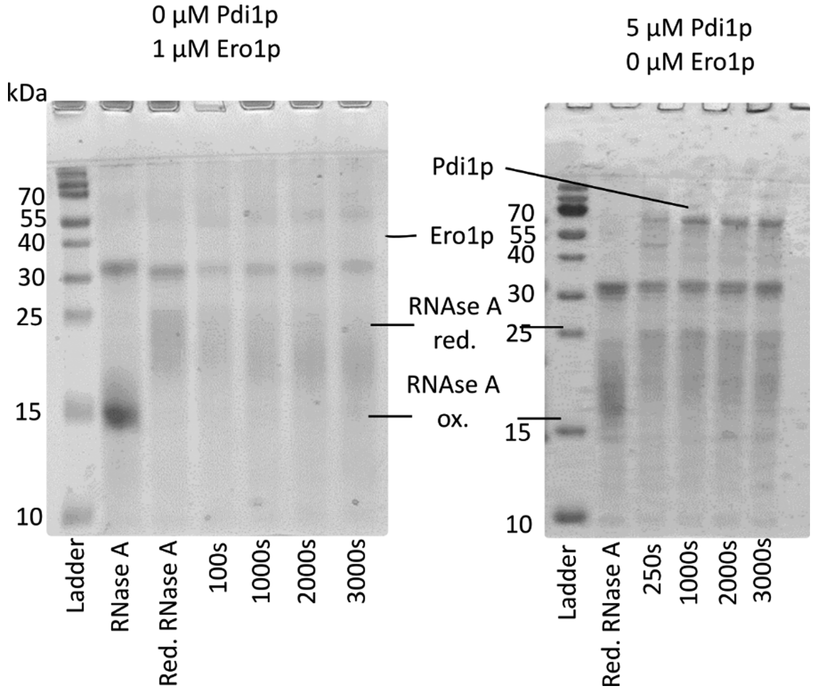

SUPPLEMENTARY FIG. S8. SDS-PAGE analysis of the oxidation of RNase A in absence of Pdi1p and Ero1p. The oxidation state of RNase A was assessed, by AMS trapping, over a similar duration to the experiments of x and S1, where either Pdi1p (*left*) or Ero1p (*right*) was not added to the reaction. *Left:* $0\,\mu M$ Pdi1p, $1\,\mu M$ Ero1p, and $60\,\mu M$ rRNase A. *Right:* $5\,\mu M$ Pdi1p, $0\,\mu M$ Ero1p, and $60\,\mu M$ rRNase. These data show that in the absence of either component (Pdi1p or Ero1p) during the timeframe of the experiment, the degree of spontaneous RNase A oxidation was minimal.
